# Pro-inflammatory-Related Loss of CXCL12 Niche Promotes Acute Lymphoblastic Leukemic Progression at the Expense of Normal Lymphopoiesis

**DOI:** 10.3389/fimmu.2016.00666

**Published:** 2017-01-05

**Authors:** Juan Carlos Balandrán, Jessica Purizaca, Jennifer Enciso, David Dozal, Antonio Sandoval, Elva Jiménez-Hernández, Leticia Alemán-Lazarini, Vadim Perez-Koldenkova, Henry Quintela-Núñez del Prado, Jussara Rios de los Ríos, Héctor Mayani, Vianney Ortiz-Navarrete, Monica L. Guzman, Rosana Pelayo

**Affiliations:** ^1^Oncology Research Unit, Mexican Institute for Social Security, Mexico City, Mexico; ^2^Molecular Biomedicine Program, CINVESTAV, IPN, Mexico City, Mexico; ^3^Biochemistry Sciences Program, Universidad Nacional Autónoma de Mexico, Mexico City, Mexico; ^4^Hospital para el Niño, Instituto Materno Infantil del Estado de México, Toluca, México; ^5^Servicio de Hematología, Hospital Pediátrico Moctezuma, SSA, Mexico City, Mexico; ^6^Department of Molecular Biomedicine, CINVESTAV, Mexico City, Mexico; ^7^Laboratorio de Microscopía, Centro de Instrumentos, Coordinación de Investigación en Salud, Centro Médico Nacional Siglo XXI, Instituto Mexicano del Seguro Social, Mexico City, México; ^8^UMAE “Dr. Victorio de la Fuente Narvaez”, Instituto Mexicano del Seguro Social, Mexico City, México; ^9^Division of Hematology and Medical Oncology, Weill Cornell Medicine, New York, NY, USA

**Keywords:** acute lymphoblastic leukemia, tumor microenvironment, CXCL12, leukemic niches, pro-inflammatory bone marrow

## Abstract

Pediatric oncology, notably childhood acute lymphoblastic leukemia (ALL), is currently one of the health-leading concerns worldwide and a biomedical priority. Decreasing overall leukemia mortality in children requires a comprehensive understanding of its pathobiology. It is becoming clear that malignant cell-to-niche intercommunication and microenvironmental signals that control early cell fate decisions are critical for tumor progression. We show here that the mesenchymal stromal cell component of ALL bone marrow (BM) differ from its normal counterpart in a number of functional properties and may have a key role during leukemic development. A decreased proliferation potential, contrasting with the strong ability of producing pro-inflammatory cytokines and an aberrantly loss of CXCL12 and SCF, suggest that leukemic lymphoid niches in ALL BM are unique and may exclude normal hematopoiesis. Cell competence *ex vivo* assays within tridimensional coculture structures indicated a growth advantage of leukemic precursor cells and their niche remodeling ability by CXCL12 reduction, resulting in leukemic cell progression at the expense of normal niche-associated lymphopoiesis.

## Introduction

The increasing evidence of the tumor microenvironment role in carcinogenesis has substantially modified the perspective of cancer pathobiology, favoring a more integrative view that considers the convergence of cellular genetics and an abnormal microenvironment as crucial elements for the development and maintenance of the disease (1). Notably, in concordance with most types of cancer, induced chronic inflammation and editing of the microenvironment by tumoral cells have been reported in some hematological malignancies, including acute lymphoblastic leukemia (ALL) (2–5).

Acute lymphoblastic leukemia, characterized by a multiclonal uncontrolled production of lymphohematopoietic precursors, represents the most frequent childhood malignancy worldwide, showing a higher incidence in the Hispanic population and specifically, an increased ratio of high-risk patients in Mexico (6–8). Despite the notable progression in the disease management (9, 10), the emergence of mixed-lineage leukemias, chemoresistance, and minimal residual disease decreases the probabilities for therapy success and determines relapse in more than 20% of the treated patients. As observed for the normal hematopoietic progenitors, neither premalignant cells nor leukemic blasts work as independent and autonomous entities, but they are rather surrounded in all dimensions by bone marrow (BM) niche components that provide regulatory cues essential for their cell fate decisions such as proliferation and survival (11–14). The hematopoietic microenvironment under homeostatic conditions is a highly organized complex and dynamic network of cells [including mesenchymal stromal cells (MSC), osteoblasts, adipocytes, endothelial cells, innate immune cells, etc.] and their natural products such as cytokines, chemokines, and extracellular matrix forming a supportive scaffold for hematopoiesis (4, 5). Based on their major non-hematopoietic cellular components, at least three distinct BM hematopoietic stem cell niches have been identified: the endosteal niche, shaped by osteoblasts lining the bone surface at the endosteum, the vascular niche formed with endothelial cells, and the perisinusoidal reticular niche built by a heterogeneous population of networking stromal cells with long processes and abundant expression of CXCL12 and leptin receptor (4, 15–17). Remarkably, CXCL12-expressing osteoblasts and perivascular stromal cells are critical for the earliest stages of the lymphoid program (18, 19) and may additionally provide FLT3L, SCF, and IL-7 that are essential factors for development of B-lymphoid progenitor and precursor cells. Depletion of CXCL12 in the stromal cells that constitute the lymphoid niche critically diminishes commitment and differentiation of B cell progenitors (18). An overlap existing between CXCL12-SCF- and IL-7-producing cells has been recently suggested (20, 21).

In the initial stage of leukemogenesis, normal and malignant populations apparently share BM niches. However, it is becoming clear that progressive changes in both, cell composition and function of the microenvironment may govern abnormal progenitor cell activity and drive to establishment of exclusive structures suitable for leukemic cell proliferation but incompatible for sustaining normal hematopoiesis, that ultimately lead to disease (4, 22–26). Previous studies in mice models and leukemia patients revealed that some of the BM microenvironmental alterations include an increased concentration of SCF and a decreased production of CXCL12 (2, 24, 27). Furthermore, remodeling of BM niche to favor tumor progression over normal cells (a niche-dependent with niche alteration mechanism) has been recently suggested by elegant experimental chronic myeloid leukemia models where leukemic cells decreased expression of CXCL12 concomitant to increased G-CSF production, impairing normal hematopoiesis and facilitating leukemia growth (22, 28). Of note, a similar scenario has been documented for xenografted ALL precursor cells that disrupt the normal niches by downregulation of CXCL12 and further exclusion of normal HSC from their natural niche (24).

Accordingly, previous work in our lab suggests an important damage in the frequency and function of hematopoietic stem and progenitor cells (HSPC) in the BM of ALL patients accompanied by an abnormal and pro-inflammatory secretion profile (e.g., increased secretion of IL-1β and TNFα) (29, 30). In order to address the molecular pathways involved in hematopoietic and microenvironmental alterations that may favor disease progression, we recently constructed a Boolean model, which demonstrates that constitutive activation of NF-κB in the hematopoietic molecular network was sufficient to establish a positive feedback loop that maintain the constitutive secretion of pro-inflammatory cytokines (e.g., IL-1β and G-CSF) and induce the disruption of the CXCR4/CXCL12 axis, which is crucial for the maintenance of the hematopoietic cells in their regulatory niche. Interestingly, this constitutive activation also induces the upregulation of the alternative CXCL12 receptors that may activate additional mechanisms to make front to pro-inflammatory insults (31). Thus, both, experimental and theoretical observations strongly indicate a fundamental role of MSC and CXCL12 expression levels in leukemia progression.

Our findings now suggest that the expression of CXCL12 in BM lymphoid niches under pro-inflammatory settings is critically reduced in ALL, allowing the malignant cell dominance over normal differentiation.

## Materials and Methods

### Isolation of Normal and Leukemic Cells from BM

Bone marrow specimens, collected according to international and institutional guidelines, were obtained through BM aspiration from 26 pediatric patients referred to the “IMIEM” Children’s Hospital (Toluca, Mexico) and to the “Moctezuma” Children’s Hospital (Mexico City, Mexico) and diagnosed with ALL, before any treatment (Table S1 in Supplementary Material). Control BM specimens were obtained from six healthy children undergoing minor orthopedic surgery. Mononuclear cells (MNC) were obtained by Ficoll-Paque (GE Healthcare) density gradient centrifugation and then enriched in the CD34^+^ fraction using the human CD34 MicroBead Kit UltraPure human (MiltenyiBiotec) according to the manufacturer’s instructions. Cell purity and yield were measured by flow cytometry using a BD FASCanto II. All procedures were approved by the Ethics, Research, and Biosafety Committees at the participant hospitals and by the National Committee of Scientific Research at the Mexican Institute for Social Security (CIEICE-007-01-13, R-2012-3602-29, and R-2015-785-120). All the samples were collected after written informed consent from the parents.

### Isolation and Expansion of BM MSC

Mononuclear cells (2–4 × 10^6^) from normal or leukemic BM were placed in culture with low glucose Dulbecco’s modified Eagle’s medium (DMEM, Gibco) supplemented with 10% fetal bovine serum (FBS, Gibco) and 100 U/ml of penicillin/streptomycin (Gibco). MSC were then isolated by their plastic adherence properties according to the International Society for Cellular Therapy (23). Upon confluence, cell monolayers were trypsinized and reseeded for their expansion and biological characterization for at least one more week. All experiments were performed with harvested cells from the third or fourth passage. Immunophenotype of MSC was confirmed by flow cytometry as CD45^−^CD34^−^HLA-DR^−^CD90^+^CD73^+^CD105^+^.

### Multi-Lineage Differentiation Capacity of MSC

To evaluate the chondrogenic differentiation potential of normal and ALL-MSC, they were cultured with chondrogenic medium [DMEM-alpha glucose, supplemented with 0.1µM dexamethasone, 50 µg/ml ascorbic acid, 110 µg/ml sodium pyruvate, 40 µg/ml prolin, 10 ng/ml TGF-β1, 50 mg/ml ITS+ premix (Becton Dickinson), 6.25 µg/ml insulin, 6.25 µg/ml transferrin, 6.25 ng/ml selenic acid, 1.25 mg/ml bovine serum albumin, and 5.35 mg/ml linoleic acid] followed by Alcian blue staining after 14 days. For adipogenic differentiation, MSC were grown in medium supplemented with 0.5mM isobutil-metilxantin, 1µM dexametasone, 10µM insulin, and 200µM indomethacin, before red oil staining. Osteogenic differentiation was investigated using low glucose DMEM medium supplemented with 15% of osteogenic supplement, 10^−7^M dexamethasone, and 0.2 mM ascorbic acid. Cells were grown during 3 weeks and then stained with von Kossa dye.

### Colony-Forming Units-Fibroblast (CFU-F) Assay

Mesenchymal stromal cells’ progenitor frequencies were recorded by fibroblast colony formation assay. Crescent numbers of MNC were plated in DMEM supplemented with 10% of FBS and 14 days later Wright–Giemsa staining was performed to determine the CFU-F content.

### MSC Proliferation Assay

A carboxifluorescein (CFSE) dilution assay was conducted using 10mM CFSE per 5 × 10^5^ MSC and dye dilution evaluated by flow cytometry at 24 and 48 h.

### RT-PCR Analyses for MSC Differentiation

Transcription of differentiation-related genes was evaluated by RT-PCR using the following primers: PPARγ2, Runx2, Sox9, CXCL12, HIF-1α, and IL7, with the 18s ribosomal RNA as housekeeping control. cDNAs were subject of amplification by RT-PCR (95°C initial denaturalization for 5 min, 35 amplification cycles of 5 s at 95°C, and aligning temperature in function of every single gene during 30 s and 72°C during 10 min) in a thermal cycler T100 ™BIORAD. AlphaView software (FluorChem System) was used for analysis. Primer sequences: *Runx2*: F: 5′-CACCATGTCAGCAAAACTTCTT-3′; R: 5′-TCACGTCGCTCATTTTGC-3′. *Sox9*: F: 5′-GTACCCGCACTTGCACAAC-3′; R: 5′-TCGCTCTCGTTCAGAAGTCTC-3′. *PPARG2*: F: 5′-TCCATGCTGTTATGGGTGAA-3′; R: 5′-TGTGTCAACCATGGTCATTTC-3′. *CXCL12*: F: 5′-CCAAACTGTGCCCTTCAGAT-3′; R: 5′-CTTTAGCTTCGGGTCAATGC-3′. *18s*: F: 5′-AAATCCAGCGCAGTACAAGATCCCA-3′; R: 5′-TTTCTTCTTGGACACACCCACGGT-3′.

### Lymphoid Differentiation Coculture System

Around 1,000 lymphoid progenitors were seeded (input value) in alpha-MEM 10% FBS supplemented with 100 ng/ml rhSCF, 100 ng/ml rhFlt3-L, 20 ng/ml rhIL-7, and 10 ng/ml rhIL-15. Cocultures were maintained during 3–6 weeks, and B precursor cells production was evaluated by multiparametric flow cytometry (FACSCalibur, BD). Results were recorded as yield per input (absolute number of B cell precursors that are produced per each initial progenitor).

### Flow Cytometry Analyses of Cell Populations

#### Hematopoietic Cells

CD34^+^ cells from normal bone marrow (NBM) and ALL were enriched using the Human CD34 Progenitor Cell Isolation Kit (Miltenyi Biotec) according to the manufacturer’s instructions. After staining with PE-conjugated anti-lineage markers (CD3, CD8, TCR, CD56, CD14, CD11b, CD20, CD19, and CD235a) and anti-CD34-APC, primitive cell populations were highly purified by multicolor flow cytometry using a FACSAria sorter (BD Biosciences). HSPC were separated as Lin-CD34^+^. Upon harvesting from culture, anti-CD56 and anti-CD19 antibodies were used to evaluate cell production in lymphoid lineage conditions. Pro-B blast population was identified as CD34^+^CD19^+^, while Pre-B cells as CD34^−^CD19^+^.

#### Mesenchymal Stromal Cells

Immunophenotyping was conducted by surface staining of MSC with 1:100 dilution antibodies, while intracellular staining was performed by incubating primary CXCL12 and HIF-1α antibodies (1:100 dilution) during 2 h at 4°C in the dark upon cell fixation and permeabilization using Cytofix/Cytoperm (BD Biosciences) solution, according to the manufacturer’s instructions. After cell washing, the appropriate secondary antibody (1:1,000 dilution) was incubated for 1 h at 4°C in the dark. Cells were washed, and the pellets resuspended prior to flow cytometry analysis. Analysis of flow cytometry data was performed using the FlowJo 7.6.1 software (TreeStar Inc., Ashland, OR, USA).

### Antibodies

Most primary and secondary antibodies came from Abcam: mouse monoclonal CXCL12 (MM0211-9N26), rabbit recombinant monoclonal SCF (EP665Y), rabbit polyclonal HIF-1α (AB103063), or mouse monoclonal connexin-43 (Cx-43) (4E6.2). Goat Anti-Mouse Alexa Fluor^®^ 488 (IgG H&L) (ab150113), Goat Anti-Rabbit Alexa Fluor^®^ 405 (IgG H&L) (ab175652), and Donkey Anti-Mouse Alexa Fluor^®^ 405 (IgG H&L) (ab175658). Rabbit Anti-Human phospho-NF-kB p65 (ser536) (93H1) came from cell signaling. Source for immunophenotyping antibodies: CD34-APC (Clone 581, BioLegend), CD19-FITC (Clone HIB19, BioLegend), CD45-PECy5 (Clone HI30, BioLegend), HLA-DR-PE (clone L243, BioLegend), CD90-APC (5E10, BD), CD73-PE (AD2, BD), and CD105-FITC (266, BD).

### Cytokines Detection

Supernatants were removed from normal and ALL-MSC cultures and the secreted factors quantified by multiplex cytokine detection assay (Milliplex Map, Millipore) according to the manufacturer’s instructions.

### Induction of Hypoxic Conditions

Mesenchymal stromal cells were treated with 100µM cobalt chloride II (CoCl_2_) during 24 h. Cytoplasmic stabilization of HIF-1α was measured by flow cytometry and its nuclear translocation by indirect immunofluorescence microscopy.

### Hematopoietic Proliferation by BrdU Incorporation Assay

Mesenchymal stromal cells–hematopoietic cell cocultures were supplemented with BrdU during 24 h and leukemic cells harvested, fixed, and permeabilized. DNAse treatment was performed for 1 h at 37°C before cell incubation with anti-BrdU. Proliferation rates were analyzed by multiparametric flow cytometry.

### Indirect Immunofluorescence Microscopy

Mesenchymal stromal cells were fixed with paraformaldehyde 4% and permeabilized with PBS–Triton 0.01%. Fc receptors were blocked with 3% PBS–FBS, and cells were incubated overnight with anti-CXCL12, anti-SCF, anti-HIF-1α, anti-IL-7, anti-Cx-43, or anti-phospho-p65 monoclonal antibodies. AlexaFluor^®^488 and AlexaFluor^®^405 conjugated antibodies were further added. Nuclei were stained with propide iodide before cell preparations were analyzed in a confocal microscope (NIKON). Images were analyzed using the ImageJ 1.51h software (Wayne Rasband, NIH, USA).

### Tridimensional (3D) Coculture System

Mesenchymal stromal cells were induced to form spheroids in non-adherent wells and leukemic precursor cells cocultured with each spheroid. Normal or leukemic cells were labeled with fluorescent dyes CellTraceViolet^®^ or CellTrace Far Red^®^ (Life Technologies) to distinguish each other by confocal microscopy throughout competence assays. Immunophenotype of leukemic cells were performed by flow cytometry after spheroid enzymatic digestion. CXCL12 expression was concomitantly evaluated by confocal microscopy. Briefly, spheroids were washed several times with PBS followed by fixing and permeabilization for 30 min. Spheroids were incubated with antibodies as described before.

### Statistics

Prism V3.02 (GraphPad) software was used to perform statistical data analysis. Yield per input results were showed as media values ± SD. Differences within groups were established by non-parametric test Mann–Whitney, considering significant probability values <0.05. For three or more groups, the Kruskal–Wallis test has been applied. Comparative analyses are reported as Mean ± SEM.

## Results

### Functional Alterations in MSC Set Up a Pro-inflammatory Microenvironment in ALL

**Figure 1:**
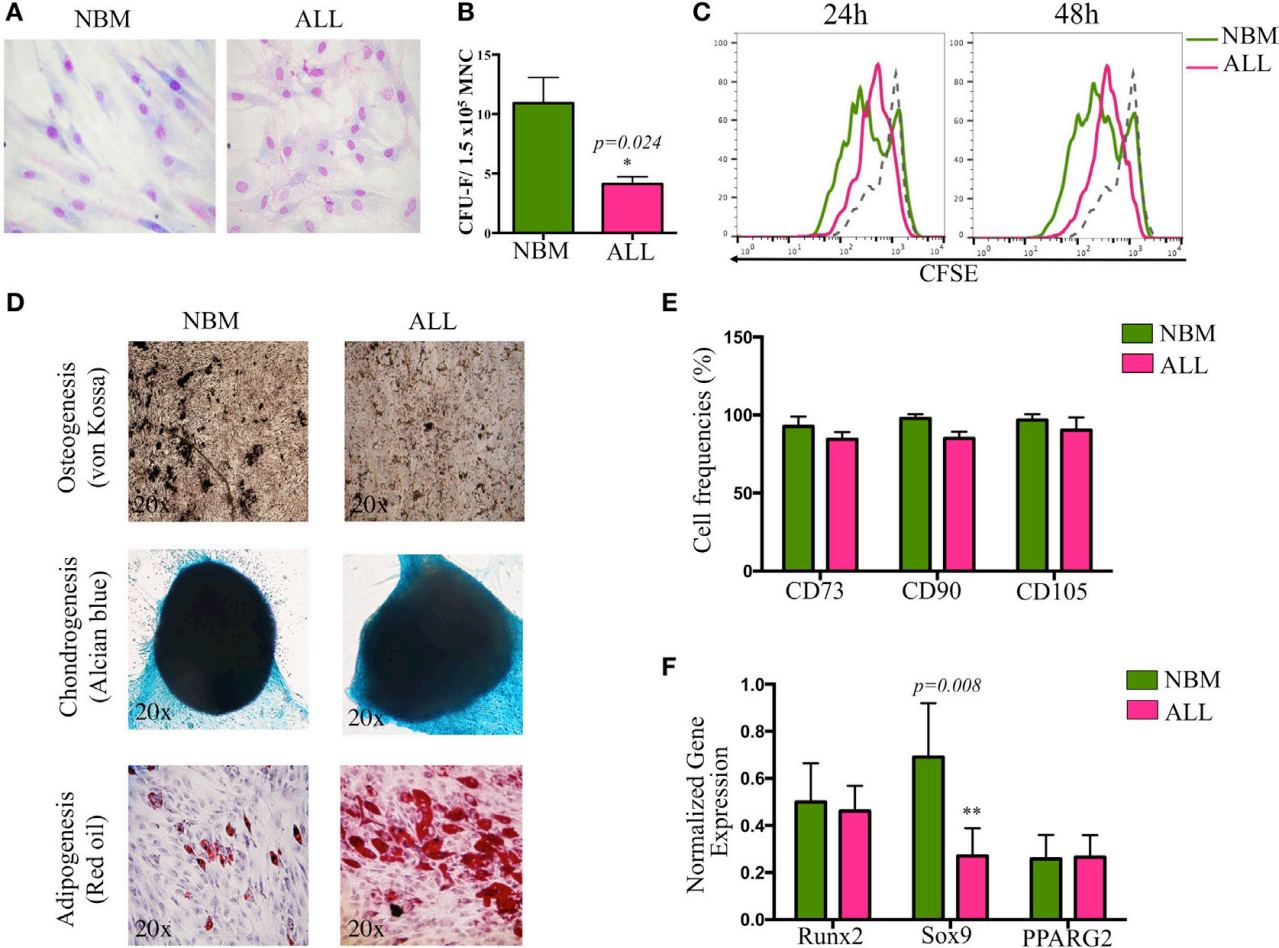
**Biological characterization of mesenchymal stromal cells (MSC) derived from bone marrow of ALL patients**. MSC were evaluated according to morphology **(A)**, colony-forming unit-fibroblast capacity **(B)**, proliferation rates by carboxifluorescein dilution assay **(C)**, multiple differentiation potential (*N*_NBM_ = 5, *N*_ALL_ = 7) **(D)**, minimal criteria immunophenotype (*N*_NBM_ = 5, *N*_ALL_ = 7) **(E)**, and differentiation genes expression (*N*_NBM_ = 4, *N*_ALL_ = 5) **(F)**. NBM, normal bone marrow; ALL, acute lymphoblastic leukemia.

Bone marrow MSC from childhood ALL patients were characterized according to their morphological, phenotypical, and functional properties established as the minimal criteria by the International Society for Cellular Therapy (32). No differences in morphological aspects between cells from both sources were apparent. However, ALL-MSC showed intercellular disorganization when monolayer structures were observed (Figure 1A). Remarkably, although CFU-F capacity and cell proliferation were reduced (Figures 1B,C), increasing proliferation activity was recorded when 7-day cultures were performed, suggesting that once the ALL primary MSC are adapted to *in vitro* culture conditions, autocrine growth factors may induce their high extent proliferation. Nevertheless, simple population dynamics assays by cell counting suggest ALL-MSC have no ability to reach normal rates (Figure S1 in Supplementary Material). In contrast, neither their differentiation potential to chondrogenic, adipogenic, and osteogenic lineages nor their phenotypes were truly compromised (Figures 1D,E). As known, although specific MSC markers are still under investigation, currently the most accepted phenotype is CD34^−^CD45^−^CD14/CD11^−^CD90^+^CD73^+^CD105^+^ with variable expression of MHC II, CD29 (integrin β1), CD49b (integrin α2), CD44 (HCAM), CD54 (ICAM-1), CD58 (LFA-3), and CD166 (ALCAM). Here, our data indicate a correspondence in the phenotype of the MSC from ALL and NBM, showing minor differences in expression of HLA-ABC, CD13, CD73, CD140b, CD44, CD54, CD49b, and CD166, which may relate to potential abnormalities in the capacity of cell–cell intercommunication with leukemic precursors within the BM. Interestingly, as previously reported for angiogenic-stimulated abnormal MSC in hematological malignancies (33), CD90 was reduced in a subpopulation of ALL (Figure 1E; Figure S1 in Supplementary Material). Whether this molecule may discriminate more primitive stages of mesenchymal linage, as it does in the hematopoietic system, remains elusive. Differentiation gene transcripts, including Runx2 (osteogenesis), Sox9 (chondrogenesis), and PPARG2 (adipogenesis) revealed a substantial reduction in Sox9 expression by ALL cells, suggesting altered lymphopoiesis associated to defects in osteochondrogenesis (Figure 1F) as reported (34). Despite the fact that PPARG2 expression was not obviously altered, a number of ALL BM samples displayed augmented ability to differentiate toward adipocytes (Figure 1D).

**Figure 2:**
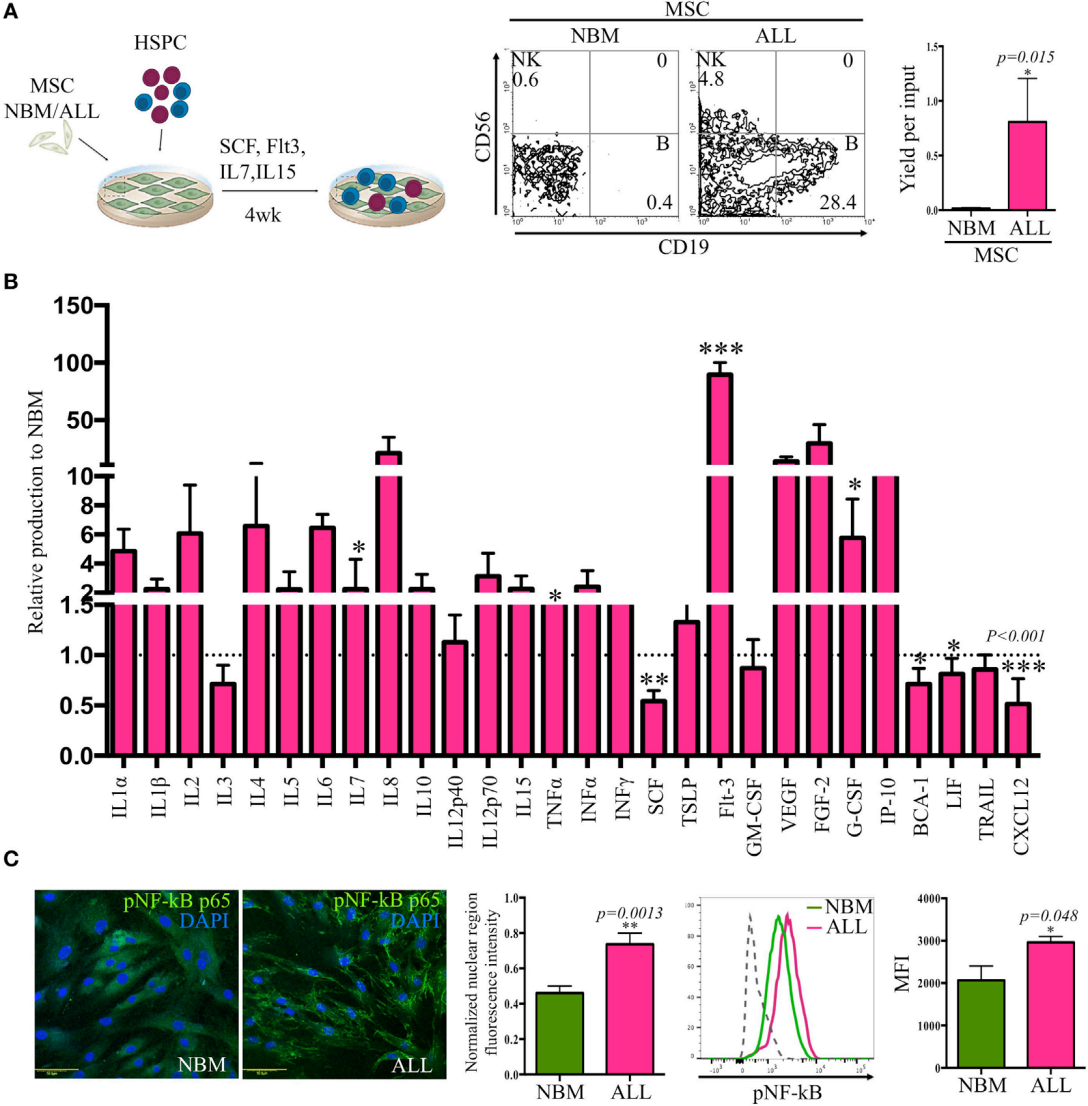
**Acute lymphoblastic leukemia (ALL)-mesenchymal stromal cells (MSC) contribute to leukemic cell maintenance by creating a pro-inflammatory microenvironment**. Hematopoietic stem and progenitor cells (HSPC) from ALL bone marrow were cocultured on normal bone marrow (NBM)-MSC or ALL-MSC monolayers in lymphoid conditions during 4 weeks before flow cytometry analysis of the newly produced cells, and the B cell yield per input were recorded (*N*_NBM_ = 5, *N*_ALL_ = 7) **(A)**. Cytokines, chemokines, and growth factors production by NBM and ALL-MSC were evaluated after collection of 24-h supernatants. Data are normalized to NBM-MSC secretion **(B)** (*N*_NBM_ = 5, *N*_ALL_ = 13). Phosphorylated p65 (NF-κB) was evaluated by immunofluorescence microscopy (*N*_NBM_ = 3, *N*_ALL_ = 4) [**(C)**, left panel] and flow cytometry [**(C)**, left panel] of MSC (*N*_NBM_ = 3, *N*_ALL_ = 5). Normalized nuclear fluorescence intensity and mean fluorescence intensity (MFI) from flow cytometry are shown.

Notably, the hematopoietic support capability of MSC from ALL BM was increased when compared to their normal counterparts (Figure 2A), though it is uncertain the normal or leukemic origins of such supported cells. When normal primitive hematopoietic cells are placed onto ALL-MSC monolayers to support cell differentiation, the production of CD19^+^ B cells and CD56^+^ NK cells is critically impaired (evaluated as cell frequencies and as yield per input progenitor). In contrast, near 30 and 15% of the produced cells are CD19^+^ B-lineage or CD56^+^ NK cells, respectively, in normal to normal conditions (Figure S2 in Supplementary Material). Together, the data may strengthen the notion that abnormal MSC from ALL BM constitute special niches convenient for leukemic cell proliferation and/or differentiation but unable to sustain normal hematopoietic development (Figure S3 in Supplementary Material). Moreover, we extended these studies to explore the ALL-MSC ability of intercommunicating to HSPC by evaluating the display of the major adhesion VLA-4/VCAM-1 axis and found a substantial reduction of VLA-4 expression by ALL hematopoietic MNC, as well as decreased VCAM-1 expression by ALL-MSC. Thus, suboptimal adhesion and interplay between hematopoietic and mesenchymal components of the ALL BM may contribute to malignant cell maintenance at the expense of normal hematopoiesis (Figure S2 in Supplementary Material).

At least 16 soluble factors relevant to lymphoid development were aberrantly secreted, as supernatant contents indicated high levels of pro-inflammatory cytokines IL-1α, IL-6, IL-12p70, and TNFα, as well as interferon type I and type II and early growth factors like Flt3, G-CSF, and IL-7 (Figure 2B). Remarkably, CXCL12 (stromal derived factor-1) and SCF (stem cell factor), two components of the hematopoietic microenvironment and main regulators of stem cell maintenance that have shown to be key players in the early lymphopoiesis pathway, were critically reduced. Accordingly, ALL-MSC-conditioned medium contained high amounts of G-CSF, which has shown the ability of mobilizing hematopoietic cells from BM through a CXCL12/CXCR4 axis breaking mechanism. The observed pro-inflammatory profile was supported by the nuclear NF-κB translocation in ALL-MSC (Figure 2C). Our findings suggest a pro-inflammatory microenvironment contributed by activated MSC that may impact normal and leukemic developmental dynamics.

### Loss of CXCL12 Marks the Leukemic Niche within the BM

**Figure 3:**
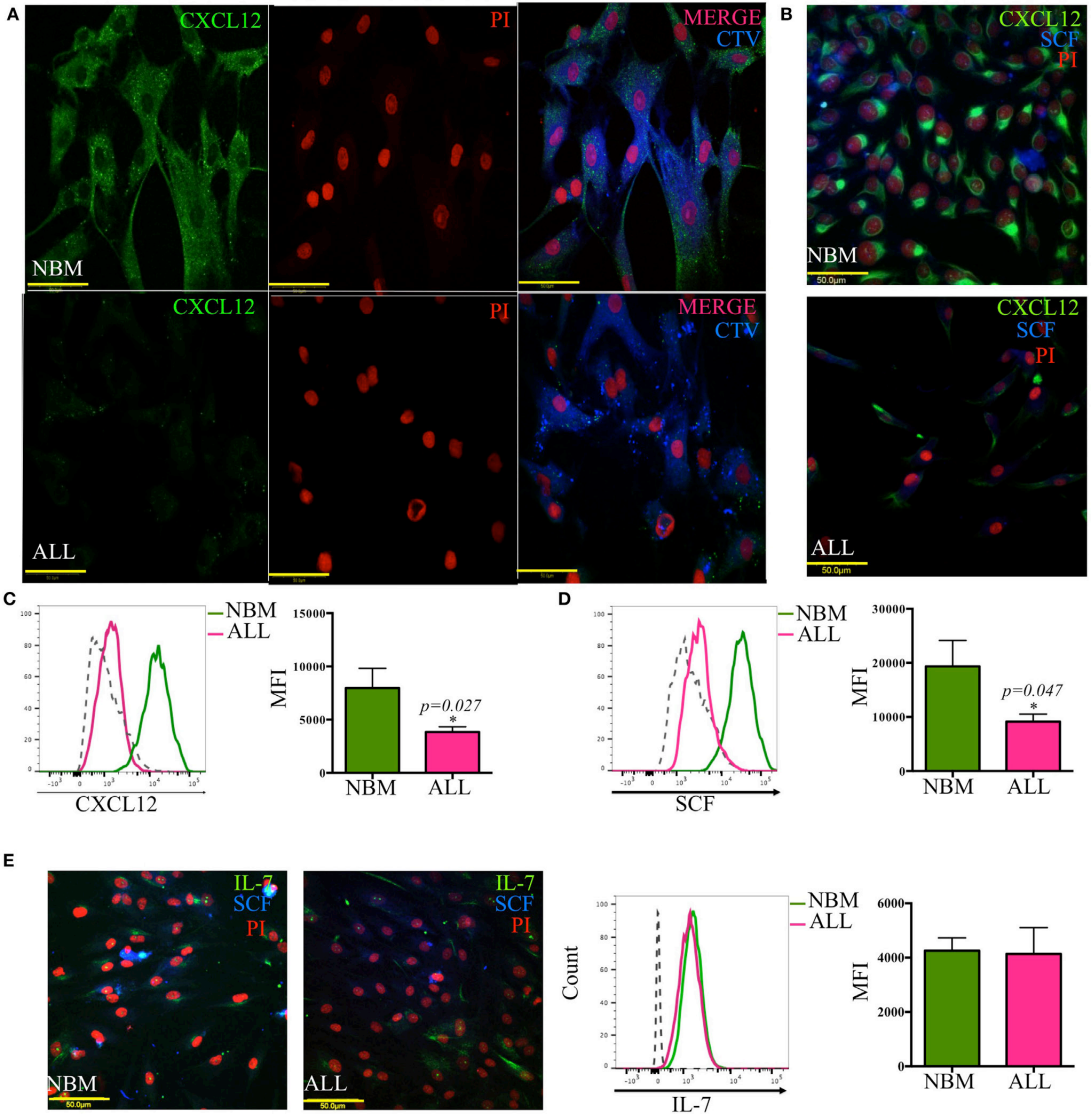
**Abnormal CXCL12 and SCF production by mesenchymal B-lymphoid niche in acute lymphoblastic leukemia (ALL)**. CXCL12 **(A–C)**, SCF **(B,D,E)**, and IL-7 **(E)** production was evaluated by immunofluorescence microscopy and flow cytometry in mesenchymal stromal cells from normal (NBM) and ALL bone marrow (*N*_NBM_ = 6, *N*_ALL_ = 12). CTV, cell trace violet.

**Figure 4:**
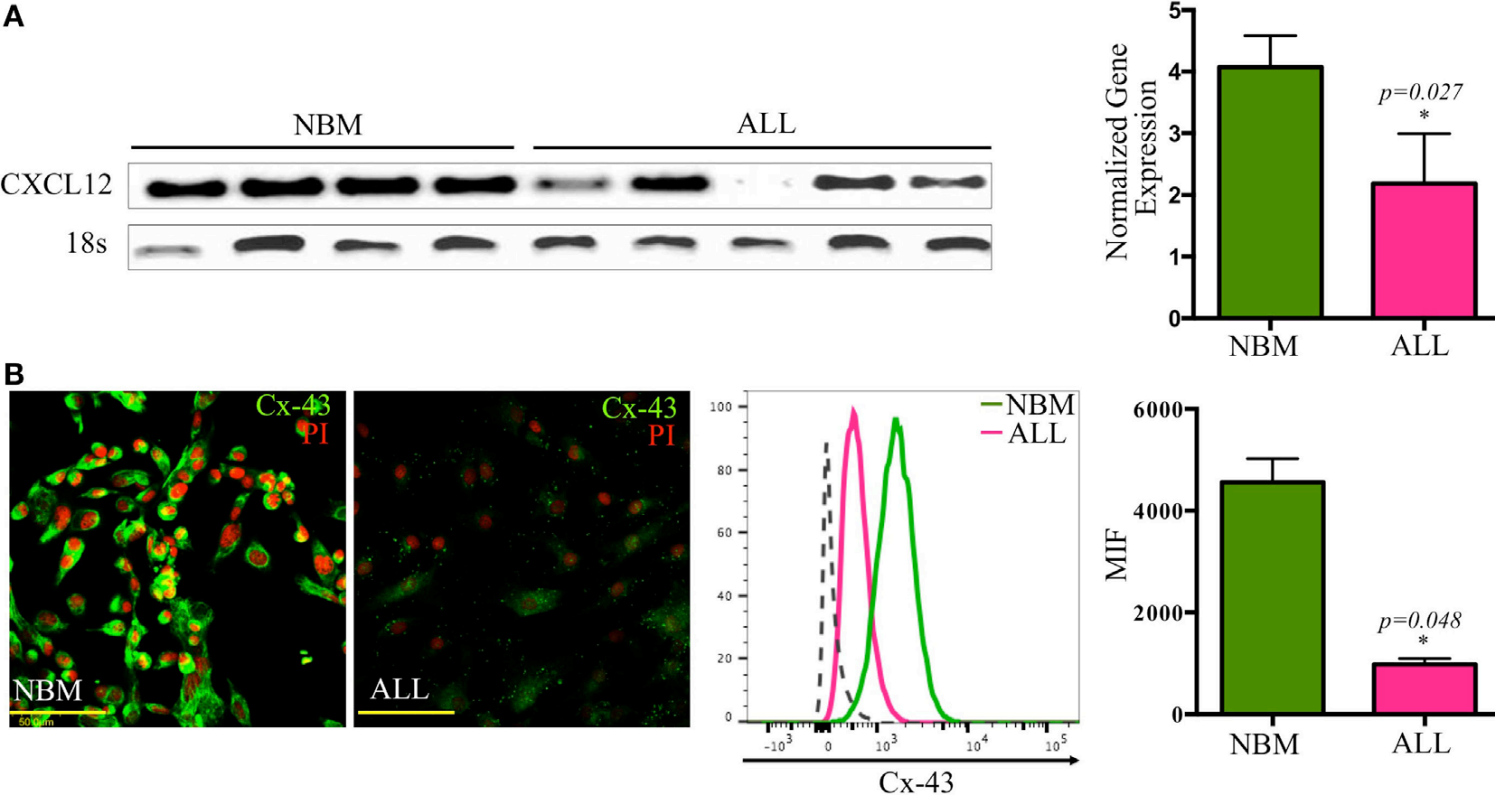
**Transcriptional and post-translational downregulation of CXCL12 in ALL**. CXCL12 mRNA expression was determined by RT-PCR in normal and ALL mesenchymal stromal cells (MSC) and their normalized gene expression tabulated (*N*_NBM_ = 4, *N*_ALL_ = 6) **(A)**. The gap-junction connexin-43 display by ALL-MSC and their normal counterpart was also analyzed by immunofluorescence microscopy and flow cytometry **(B)**. NBM, normal bone marrow; ALL, acute lymphoblastic leukemia; MFI, mean fluorescence intensity.

A crucial regulatory axis of the cross talk between lymphoid progenitors and the hematopoietic microenvironment is CXCL12/CXCR4. MSC producing high amounts of CXCL12 and SCF constitute the primary niche for B-lymphoid progenitor cells (20). Concurring with our previous supernatants observation, confocal microscopy revealed the per-cell-basis reduced production of both factors (Figures 3A–D). In contrast, IL-7 lymphoid niche appears unaltered in ALL (Figure 3E). To explore the biological mechanism associated to CXCL12 loss, we investigated the gene transcription extent and found it heterogeneously impaired among patient samples (Figure 4A). In addition, the intercommunicating stromal cell Gap-junction molecule, Cx-43, which drives secretion of CXCL12 appeared frankly downregulated in ALL-derived MSC (Figure 4B). As it is believed that in BM, MSC maintain hematopoietic cells attached to them in a semi-quiescence state through the dynamic production of CXCL12, lack of Cx-43 impacting the release of CXCL12 (35) may have important implications in architecture and function of BM niches. Moreover, homing to the endosteal niche might relate to Cx-43, due to its participation in the bidirectional traffic from BM to the periphery and *vice versa* during ALL (36).

**Figure 5:**
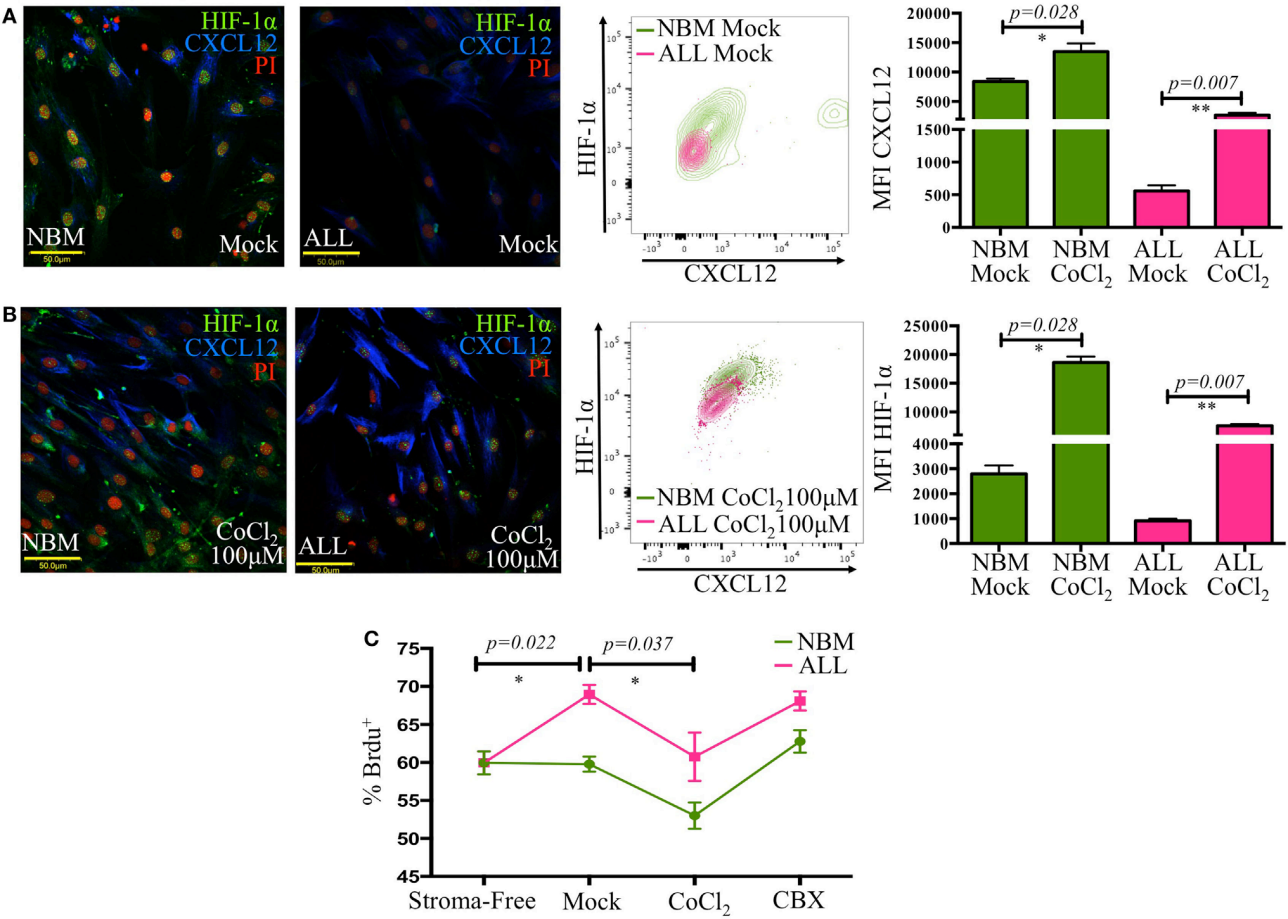
**The CXCL12 mesenchymal niche disruption is associated to an abnormal HIF-1α regulation**. HIF-1α and CXCL12 displayed by mesenchymal stromal cells (MSC) from normal or acute lymphoblastic leukemia (ALL) bone marrow were evaluated by immunofluorescence microscopy and flow cytometry upon standard cultures [**(A)**, left panel] or hypoxic-like conditions promoted by CoCl_2_ treatment for 2 h [**(B)**, left panel]. HIF-1α increase was monitored by flow cytometry [**(A,B)**, middle panels]. Mean fluorescence intensity values are shown in bar graphs [**(A,B)**, right panels]. Primary ALL cells were cocultured with normal bone marrow (NBM)- or ALL-MSC previously treated with CoCl_2_ or carbenoxolone to regulate CXCL12 expression. The culture system was pulsed with bromodeoxyuridine (BrdU), and its incorporation by leukemic cells was evaluated by flow cytometry **(C)** (*N*_NBM_ = 4, *N*_ALL_ = 5).

HIF-1α is a major transcriptional regulator of CXCL12 (37). Here, a critical reduction of its expression with a cytoplasmic localization instead of the typical nuclear translocation seen in their normal counterpart was recorded in ALL-MSC (Figure 5A). Furthermore, experimental hypoxia was not able to increase HIF-1α expression to normal levels. Chemical imitation of hypoxia by Chloride Cobalt (CoCl_2_) has been reported to create a hypoxic-like condition by disturbing the constant proteasomal degradation of HIF-1α and allowing its cytoplasmic accumulation and further nuclear translocation even during normoxic settings. Although expression of CXCL12 showed a slight boost, the end balance was never as high as from NBM (Figure 5B; Figure S4 in Supplementary Material).

Thus, in ALL, the CXCL12-dependent lymphoid niche might be intrinsically altered due at least to an impaired transcriptional regulation by HIF-1α and to an abnormal functional activity of Cx-43.

To investigate the biological relevance of loss of CXCL12 in leukemic progression, we conducted an *in vitro* cell proliferation assay by incorporation of BrdU under distinct CXCL12 scenarios. Primary leukemic cells were treated either with CoCl_2_ to increase CXCL12 expression or with carbenoxolone (CBX), a Cx-43 inhibitor, to indirectly reduce CXCL12, and their proliferative status was analyzed (Figure 5C). Of interest, malignant proliferation extent was inversely proportional to CXCL12 expression in MSC. Cell treatment with CBX did not increase the proliferation of ALL cells over the initial mock settings, suggesting, as expected, that CXCL12 regulation may result from multiple addition elements. Since the role of CXCL12 in early lymphopoiesis has been vastly studied (18, 19), our findings suggest that CXCL12 lymphoid niche may have an additional function in normality by limiting proliferation of malignant precursor cells.

### The CXCL12^+^ Lymphoid Niche Is Remodeled by Leukemia Precursor Cells, Favoring Tumor Progression at the Expense of Normal Lymphopoiesis

**Figure 6:**
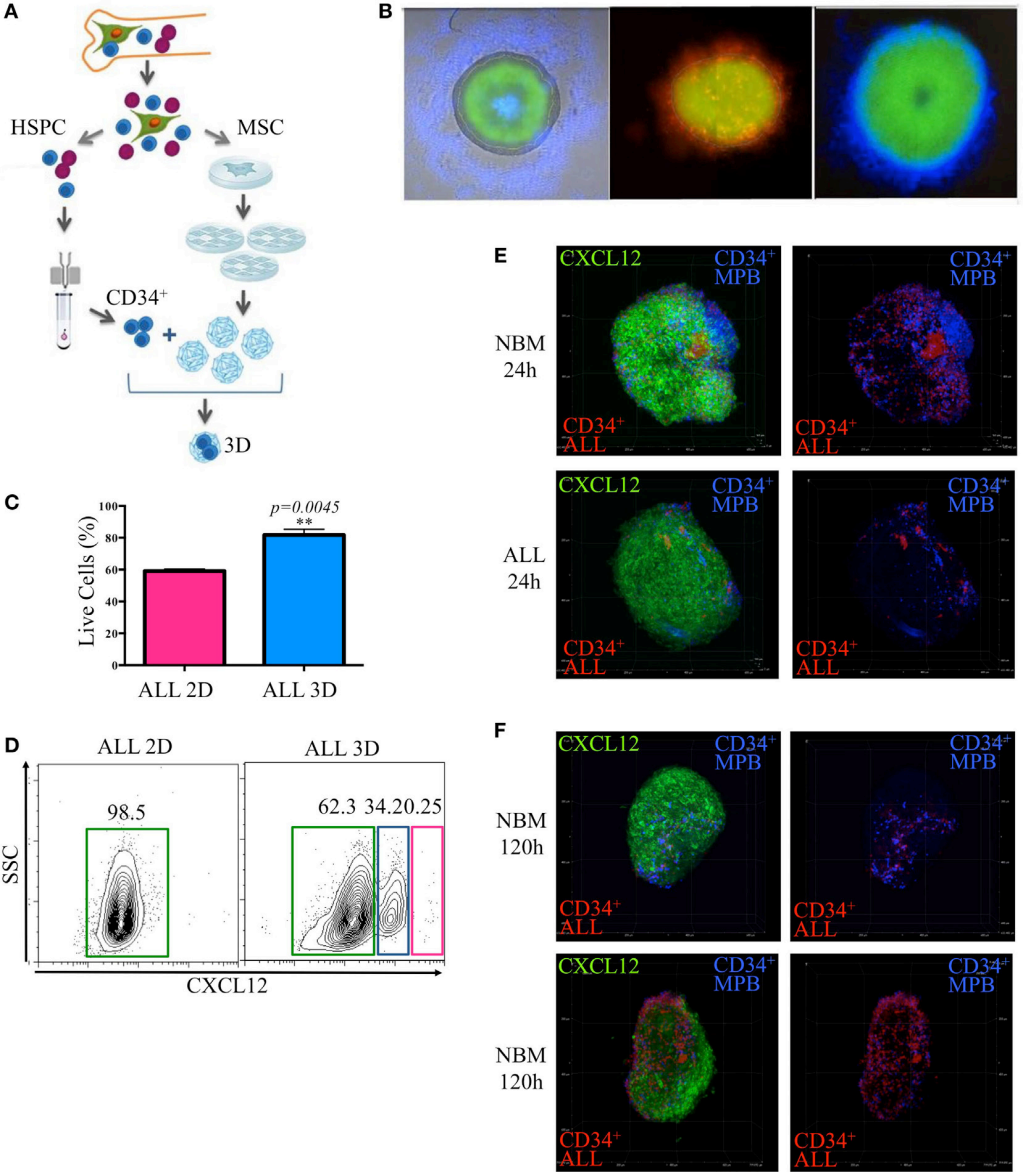
**Remodeling of the lymphoid normal niche is associated with leukemic colonization concomitant to displacement of normal progenitors**. Stromal spheroids were cocultured with acute lymphoblastic leukemia (ALL) primary cells **(A)**. The colonization was confirmed by immunofluorescence microscopy after staining CD34^+^ cells with fluorescent dyes red and blue **(B)**. Viability of leukemic cells harvested from 2D and tridimensional (3D) cocultures was assessed by flow cytometry after 7 days **(C)**. CXCL12 was evaluated by flow cytometry in 3D MSC spheroids after enzymatic digestion compared with 2D monolayer cultures **(D)**. CD34^+^ cells from mobilized peripheral blood and CD34^+^ ALL cells were cocultured in a competence ratio 1:1 for 24 h in normal bone marrow (NBM) and ALL mesenchymal stromal cells (MSC) spheroids **(E)** or during 120 h in NBM-MSC spheroids **(F)**. Spheroids were washed, and CXCL12 expression (green) was evaluated ($N_{{{\;}_{\text{NBM-CD34}}}^{+}}=2$, $N_{{{\;}_{\text{ALL-CD34}}}^{+}}=2$, *N*_NBM-MSC_ = 3, *N*_ALL-MSC_ = 4).

Because *in vitro* maintenance of primary ALL cells in conventional culture conditions and the 3D *in vivo* BM structure is critical to generate specialized hematopoietic niches, we explored the possibility of improving the microenvironmental conditions and increasing yield per input values by mimicking the structural BM properties through 3D cocultures in stromal spheroids. We have learned from this novel system that primitive normal and ALL CD34^+^ cells get the ability of colonizing them with high efficiency (Figures 6A–C) (Balandran et al., manuscript in preparation).

Population dynamics was studied by a competence assay between purified CD34^+^ cell populations from ALL BM or healthy donors mobilized peripheral blood that were previously stained with red and blue commercial fluorescent dyes, respectively.

As expected, CXCL12 was induced in 3D cultures, possibly due to reconnection of MSC and the putative hypoxia condition within the system (Figure 6D). Notably, leukemic MSC were less colonized by normal progenitors compared to the NBM counterparts at the first 24 h (Figure 6E). However, 120 h later, the normal niches were colonized by leukemic cells simultaneously to an apparent decrease of CXCL12 and displacement of normal progenitors (Figure 6F; Figure S5 in Supplementary Material).

## Discussion

Tumor cells coexist with their normal counterparts in the same microenvironment. During cancer progression, they both contend for the niche and conditioned it for the sake of tumor benefit (38–40), as has been documented for myeloid leukemias (41) or in murine models (24). CXCL12 is an essential chemokine showing constitutive expression by BM cells that directly impacts retention, quiescence, and proliferation of hematopoietic stem cells and B-cell progenitors (42). Accordingly, CXCL12 knockout mouse prenatally die due the lacking of B lymphopoiesis and myelopoiesis in the BM (43), while initiating-leukemia cells express CXCR4 (44, 45) and respond to CXCL12 by delaying apoptosis processes (46).

The cell–cell intercommunication as key factor for tumor progression has became a research priority, and increasing evidence of leukemic blasts supported by Nestin^+^ MSC indicates their ability for long-term maintenance at the disease onset (47, 48). MSC are part of the complex tissue network of the BM microenvironment (42) that establishes an essential niche for hematopoiesis (49). Here, our data suggest that ALL-MSC are defective in their proliferation and CFU-F capabilities, in concordance to observations in myeloid leukemias (50). This behavior may relate to the apparent decreased levels of Sox9 that regulates proliferation, survival, and chondrogenesis (51). Moreover, similar defects have been found in myelodysplastic syndrome patients (52, 53), in association to chromosomal abnormalities (54). Evaluating the existence of chromosomal alterations in ALL-MSC should be imperative, as it could compromise their biological functions. We have previously reported a critical decrease in the content of HSPC in BM from ALL patients and a concomitant impaired lymphoid differentiation potential (4). When the hematopoietic support is tested for ALL-MSC, B-cell differentiation is favored, suggesting that this stroma is providing the necessary signals to develop B cells. Although their origins—normal or leukemic—remain elusive, our short-term cocultures suggest a permissive role for leukemia precursor cells (data not shown).

One of the biological properties of MSC is their immune-regulatory capability mediated by TLRs and cytokine receptors (55), suggesting a dual role: anti-inflammatory and pro-inflammatory. Typically, when MSC are exposed to pro-inflammatory cytokines, including IFNγ, TNFα, IL-1α, and IL-1β, they adopt an immune-suppressive phenotype to limit inflammation previous activation. ALL-MSC expressed high levels of activated NF-κB that may result from a number of mechanisms operating together, e.g., inflammation or a VCAM-1/VLA-4 axis activation signaling (56). Interestingly, NF-κB activation *via* TNFα on MSC stimulates its osteogenic differentiation (57), but we did not find an ostegenesis-bias behavior in this study. In contrast to normal MSC that inhibit NK cytotoxic activity, recent data have shown that ALL-MSC are capable of activating NK cells and that this profile can be adopted by NBM-MSC when they are exposed to leukemia cells (58), supporting the notion of local activating factors that promote a feedback loop between MSC and leukemic precursors.

Secretion and transcription of CXCL12 by MSC is regulated through gap-junctions mainly formed by connexins where Cx-43 and connexin-45 orchestrate cellular communication within BM (35). CXCL12-abundant reticular cells in the reticular niche, which express the highest levels of CXCL12, also express the highest levels of Cx-43 (35). Furthermore, although adherence properties are dependent on cell interconnections where CXCL12 functions as an indispensable anchor molecule (35), the regulation of its production in BM samples has been controversial (46, 59).

Taking together, our data suggest that the final defect in the CXCL12 production is modulated at distinct levels in ALL, which may include FGF-2-dependent miR-31 high activity (60). We do not discard the participation of additional regulatory components other than hypoxia-related HIF-1α, based on the partial reestablishment of CXCL12 in hypoxia-like conditions.

Other compensatory mechanisms could be operating through the vascular or endosteal niche in order to maintain residual normal hematopoiesis. Here, we confirmed that CXCL12 levels on MSC regulate the proliferation of leukemic cells using a coculture system with perturbed levels of CXCL12 by treatment with CoCl_2_ or by inhibition of Cx-43 (35). Our results suggest that CXCL12^hi^ niche is acting like a leukemic repressor, contrary to a leukemic niche that favors proliferation by inhibiting the leukemic cell attachment to its stroma and promoting the entrance to cell cycle (61).

The lack of suitable *in vitro* conditions to maintain primary ALL cells plus the absence of phenotypic markers to distinguish normal and leukemic cells has delayed our disease understanding. Currently, the number of reports about the benefits of using 3D cultures is growing (62), and its use to increase the survival of leukemic cells may consummate important questions. Our observations support this notion by showing a better maintenance of primary ALL cells. Moreover, this novel model may allow the study of human–human and patient–patient microenvironment interactions and further contribute to precision medicine advance.

So far our findings concur with observations in animal models of changes in adhesion molecules and production of soluble factors controlled by malignant cells (24, 63, 64). Remarkably, the cellular communication between leukemic cells and MSC through exosomes or across nanotubes (65, 66) and the disruption of the CXCR4/CXCL12 axis (28, 59), suggesting a key role for G-CSF in this phenomena, are consistent with our previous reports about production of pro-inflammatory factors by tumor cells (30), related to G-CSF and Gfi-1 (29). Accordingly, G-CSF administration in a preclinical model of ALL showed an increased tumor burden (67), and its mechanism may be operating in the niche because B cell malignances rarely expressed G-CSF receptor and are unable to respond *in vitro* to this cytokine (68). In contrast, *in vitro* normal B-cell production in a stromal-free model is more efficient when G-CSF is supplied (69).

Loss of normal hematopoiesis is a classical event during human myeloproliferative neoplasias and leukemias where the niche downregulate essential HSPC retention factors as CXCL12, SCF, LepR, Angpt1, Cdh2, Slit2, and TGF-β1 though pro-inflammatory factors secreted by leukemia-initiating cells (64).

Recent research indicates that the CXCR4/CXCL12 pathway is also involved in modulating mitochondrial activity, regulating the production of ATP and reactive oxygen species though the precise mechanism has not been elucidated (70). Incubation of leukemic cells with CXCL12 diminishes the mitochondrial activity, coherent with a repressor profile to maintain HSPC population within BM. Nevertheless, the sustained stimulus increases the mitochondrial activity and may be due to the internalization of CXCR4 or the participation of factors as TGF-β1 (71, 72). Therefore, the study of primitive populations *in vitro*, and specifically their maintenance in regulatory niches mediated by CXCR4/CXCL12 axis, requires a model that better mimics the structural complexity *in vivo*.

A mathematical model approaching has also predicted instability in this axis following TLR ligation and/or inflammation, where an aberrant expression of NF-κB contribute to create a tumor microenvironment that allows effective growing of leukemia cells (31).

**Figure 7:**
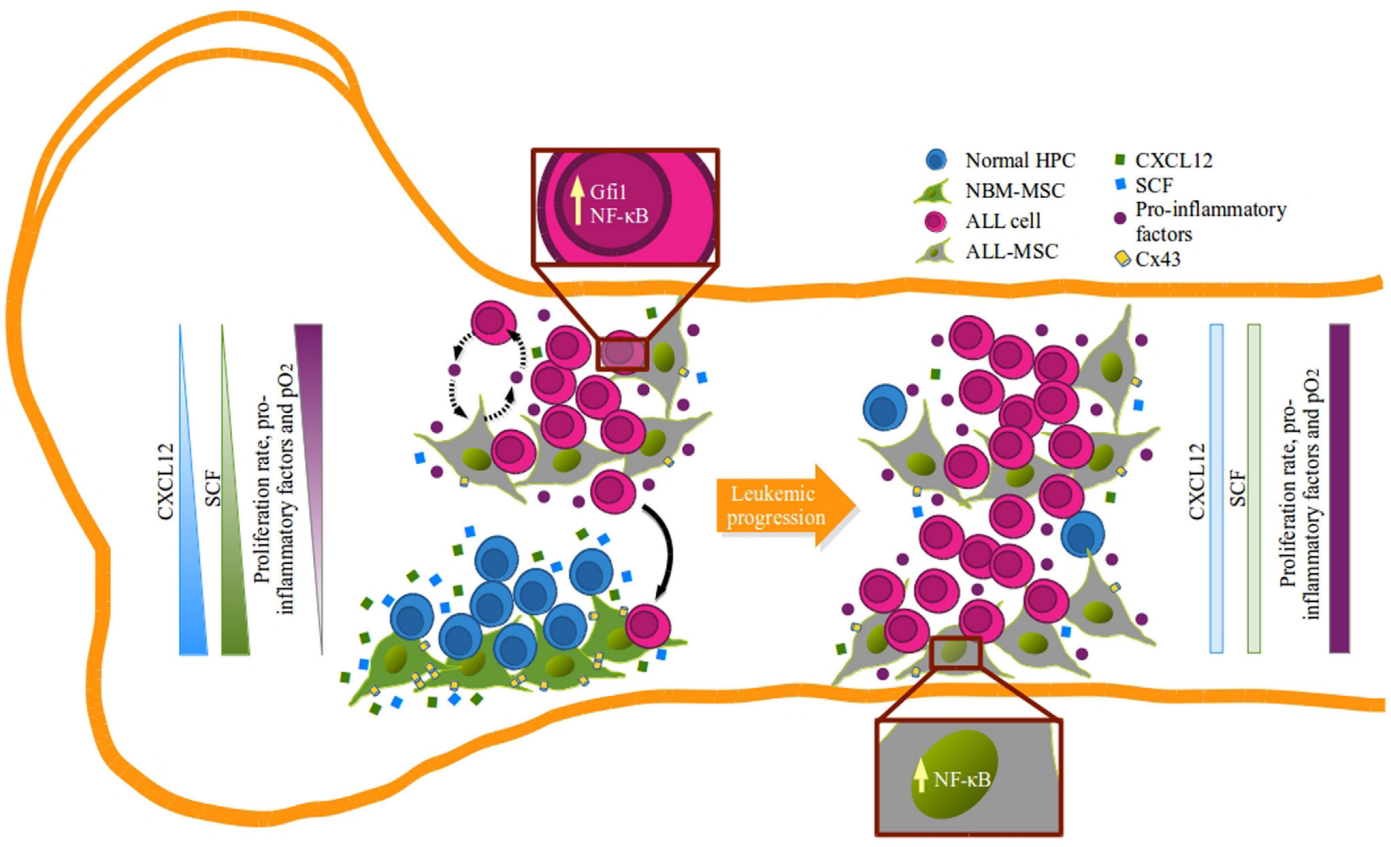
**Remodeling of B-lineage niche in acute lymphoblastic leukemia bone marrow: a model**. Suitable hematopoietic niches that support B-cell lymphopoiesis are formed by mesenchymal stromal cells (MSC) capable of producing high amounts of CXCL12 and SCF (green MSC) that may function by limiting the proliferation of lymphoid progenitors (blue) in the absence of pro-inflammatory factors and within a normal hypoxic microenvironment. During leukemogenesis settings, emerging leukemic clones (pink) hijack normal niches and progressively induce their remodeling and compromise CXCL12 and SFC expression (gray MSC). In this new sanctuary, niche edition is presumably mediated by the activation of MSC due to the pro-inflammatory elements that alter the balance and allow leukemic proliferation that result in the incompatibility of normal hematopoiesis in a feedback loop manner.

Our NBM-ALL competence data suggest the existence of two distinct niches of MSC at ALL diagnosis: one constituted by CXCL12^hi^SCF^hi^ MSC, which better support normal hematopoiesis and might be endowed with repressor functions for leukemia, and a second niche composed by remodeled MSC which CXCL12 and SCF production is abated and favors growing of malignant cells (Figure 7). Consistently, the existence of BM niches with stromal cell population expressing different levels of CXCL12 has been documented in both, normal and malignant microenvironment settings, highlighting the importance of CXCL12/CXCR4 axis in the pathobiology of hematopoietic tumors (18, 19, 22, 24, 28, 73).

Thus, during ALL progression, leukemic cells may hijack and remodel normal niches inducing alterations in hematopoiesis at central levels that displace and exhaust primitive cells *via* inflammation-derived proliferation. The design of strategies that reconstitute the main axes of normal cell-to-cell communication will be of fundamental importance.

## Ethics Statement

The Institutional Review Board approved the study, and informed consent was obtained from parents of the patients. This study was conducted with the regulations of the Ethics, Research, and Biosafety Committees at the participant hospitals and by the National Committee of Scientific Research at the Mexican Institute for Social Security (CIEICE-007-01-13, R-2012-3602-29, and R-2015-785-120).

## Author Contributions

All the authors contributed and approved the final version. Design of the study: JB, JP, JE, VO-N, MG, and RP. Biological specimens support: DD, AS, EJ-H, and HP. Experimental work: JB, JP, JE, JR, LA-L, and VP-K. Data analysis and interpretation: JB, JP, JE, HM, VO-N, MG, and RP. Final approval: JB, JE, JP, JR, DD, AS, EJ-H, LA-L, VP-K, HP, HM, VO-N, MG, and RP. JB, JP, and JE contributed equally to this work.

## Conflict of Interest Statement

The authors declare that the research was conducted in the absence of any commercial or financial relationships that could be construed as a potential conflict of interest.
