# Supplementary material for: Pro-inflammatory-Related Loss of CXCL12 Niche Promotes Acute Lymphoblastic Leukemic Progression at the Expense of Normal Lymphopoiesis
Source: Front Immunol. 2017 Jan 5;7:666. doi: 10.3389/fimmu.2016.00666 (PMC5216624; doi:10.3389/fimmu.2016.00666)
Supplement: Supplementary file 1 [file Table_1.PDF]

Supplementary Material

PRO-INFLAMMATORY-RELATED LOSS OF CXCL12 NICHE PROMOTES ACUTE LYMPHOBLASTIC LEUKEMIC PROGRESSION AT THE EXPENSE OF NORMAL LYMPHOPOIESIS

Juan Carlos Balandrán[1,2], Jessica Purizaca[1], Jennifer Enciso[1,3], Jussara Rios de los Ríos[1,3], David Dozal[4], Antonio Sandoval[4], Elva Jiménez-Hernández[5], Leticia Alemán-Lazarini[6], Vadim Perez-Koldenkova[7], Henry Quintela-Nuñez del Prado[8], Héctor Mayani[1], Vianney Ortiz-Navarrete[2], Mónica L. Guzman[9], Rosana Pelayo[1*].

***Correspondence:**
Dr. RosanaPelayo. Oncology Research Unit, Mexican Institute for Social Security. Av. Cuauhtémoc 330. Colonia Doctores, 06720 Mexico City, Mexico. Phone: +52(55)56276900 ext 22705. E-mail: rosanapelayo@gmail.com.

Table S1 | Patient Characteristics.

Patient	Age (y)	Sex	WBC $x10^3/mm3$	Phenotype	BM blasts (%)	Genetic aberrations	Risk	Risk factor
1	4	M	ND	NBM	-	-	-	-
2	7	H	ND	NBM	-	-	-	-
3	3	M	ND	NBM	-	-	-	-
4	8	M	ND	NBM	-	-	-	-
5	4	H	ND	NBM	-	-	-	-
6	8	M	ND	NBM	-	-	-	-
7	3	M	118.9	B-ALL	92.3	Neg	HR	Leukocytosis
8	14	M	108.3	B-ALL	67	t(15;17)(q22;q21) PML ex3-RARA ex5	HR	Leukocytosis
9	3	F	119.2	B-ALL	83	ND	HR	Leukocytosis
10	14	M	62.3	B-ALL	88.5	Neg	HR	Age
11	2	M	7.1	B-ALL	67	ND	SR	
12	9	M	100.7	B-ALL	81	t(1;19)(q23;p13)TCF3-PBX1(PRL)	HR	Leukocytosis
13	4	M	64.6	B-ALL	89.7	t(12;21)(p13;q22) ETV6 ex5 - RUNX1 ex4:	SR	-
14	12	F	4.9	B-ALL	72	t(1;19)(q23;p13)TCF3-PBX1(PRL)	HR	Age
15	10	M	164.5	B-ALL	85	t(9;22)(q34;q11)	HR	Leukocytosis
16	5	F	53.5	B-ALL	87.8	t(12;21)(p13;q22) ETV6 ex5 - RUNX1 ex4:	HR	-
17	9	F	55	B-ALL	86.6	t(12;21)(p13;q22) ETV6 ex5 - RUNX1 ex4:	HR	-
18	8	M	48.6	B-ALL	80	ND	HR	-
19	7	M	285.5	B-ALL	90	Neg	HR	Leukocytosis
20	15	F	58.3	B-ALL	74	Neg	HR	Age
21	7	F	107.9	B-ALL	80.6	Neg	HR	Leukocytosis
22	14	F	2.2	B-ALL	0.07	ND	HR	Age
23	7	M	33.9	B-ALL	92.2	Neg	HR	-
24	3	M	123	B-ALL	66	ND	HR	Leukocytosis
25	16	M	9.5	B-ALL	74.9	t(16;21)(p11;q22) FUS ex7 ‚Äì ERG ex13	HR	Age

y: years old; WBC: white blood cell count; BM: bone marrow; M: male; F: female; B-ALL: B cell precursor acute lymphoblastic leukemia; NBM: normal bone marrow; HR: high risk; SR: standard risk.
