# Supplementary material for: Pro-inflammatory-Related Loss of CXCL12 Niche Promotes Acute Lymphoblastic Leukemic Progression at the Expense of Normal Lymphopoiesis
Source: Front Immunol. 2017 Jan 5;7:666. doi: 10.3389/fimmu.2016.00666 (PMC5216624; doi:10.3389/fimmu.2016.00666)
Supplement: Supplementary file 2 [file Presentation_1.ppt]

## Slide 1
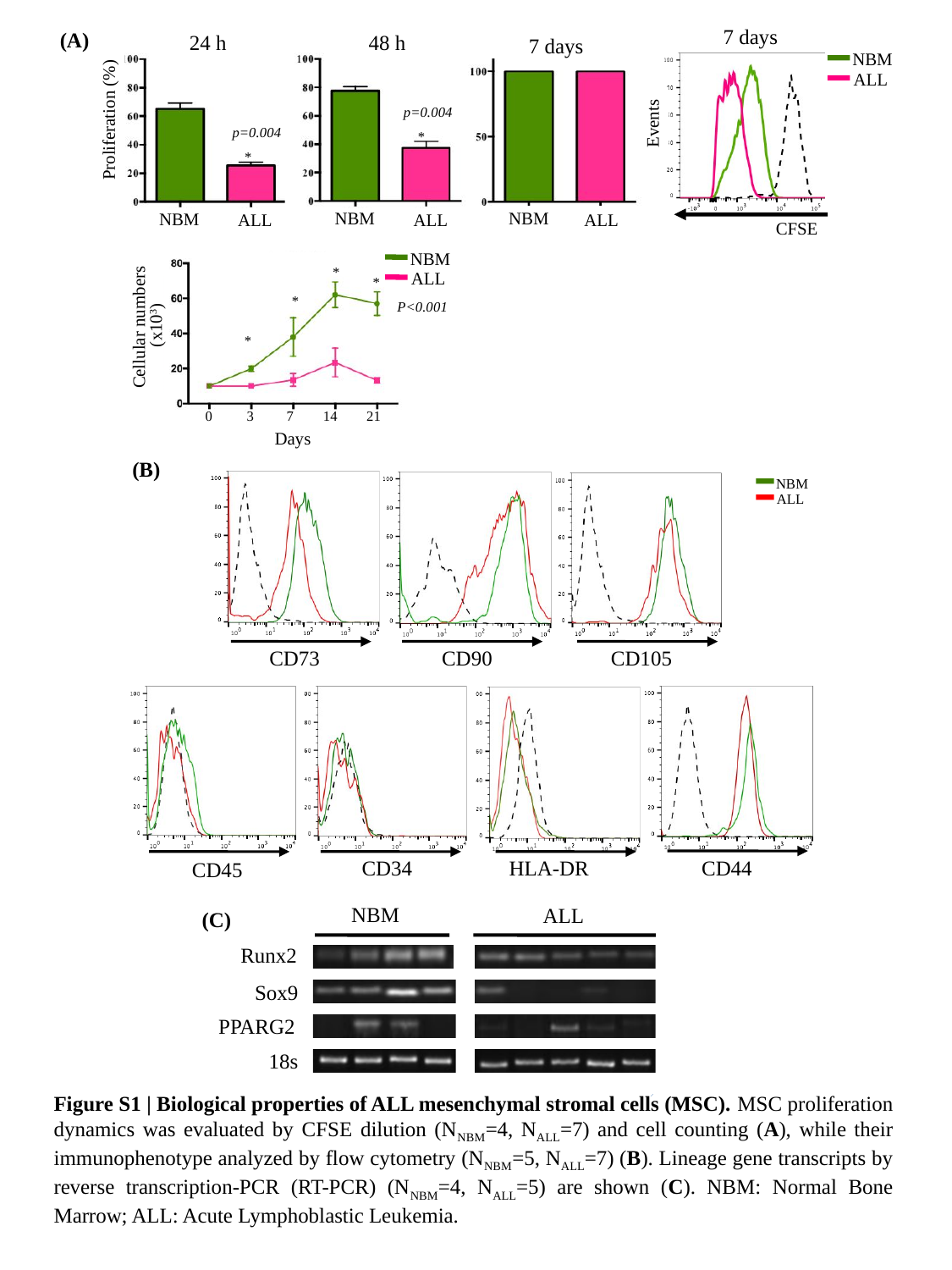

(A)
7 days
NBM
ALL
CFSE
Events
24 h
Proliferation (%)
p=0.004
*
NBM
ALL
48 h
p=0.004
*
NBM
ALL
7 days
NBM
ALL
NBM
ALL
*
*
*
P<0.001
Cellular numbers
(x103)
*
0
3
7
14
21
Days
(B)
NBM
ALL
CD73
CD90
CD105
CD34
HLA-DR
CD44
CD45
(C)
NBM
ALL
Runx2
Sox9
18s
PPARG2
Figure S1 | Biological properties of ALL mesenchymal stromal cells (MSC). MSC proliferation dynamics was evaluated by CFSE dilution (NNBM=4, NALL=7) and cell counting (A), while their immunophenotype analyzed by flow cytometry (NNBM=5, NALL=7) (B). Lineage gene transcripts by reverse transcription-PCR (RT-PCR) (NNBM=4, NALL=5) are shown (C). NBM: Normal Bone Marrow; ALL: Acute Lymphoblastic Leukemia.
