# Supplementary material for: Pro-inflammatory-Related Loss of CXCL12 Niche Promotes Acute Lymphoblastic Leukemic Progression at the Expense of Normal Lymphopoiesis
Source: Front Immunol. 2017 Jan 5;7:666. doi: 10.3389/fimmu.2016.00666 (PMC5216624; doi:10.3389/fimmu.2016.00666)
Supplement: Supplementary file 3 [file Presentation_2.ppt]

## Slide 1
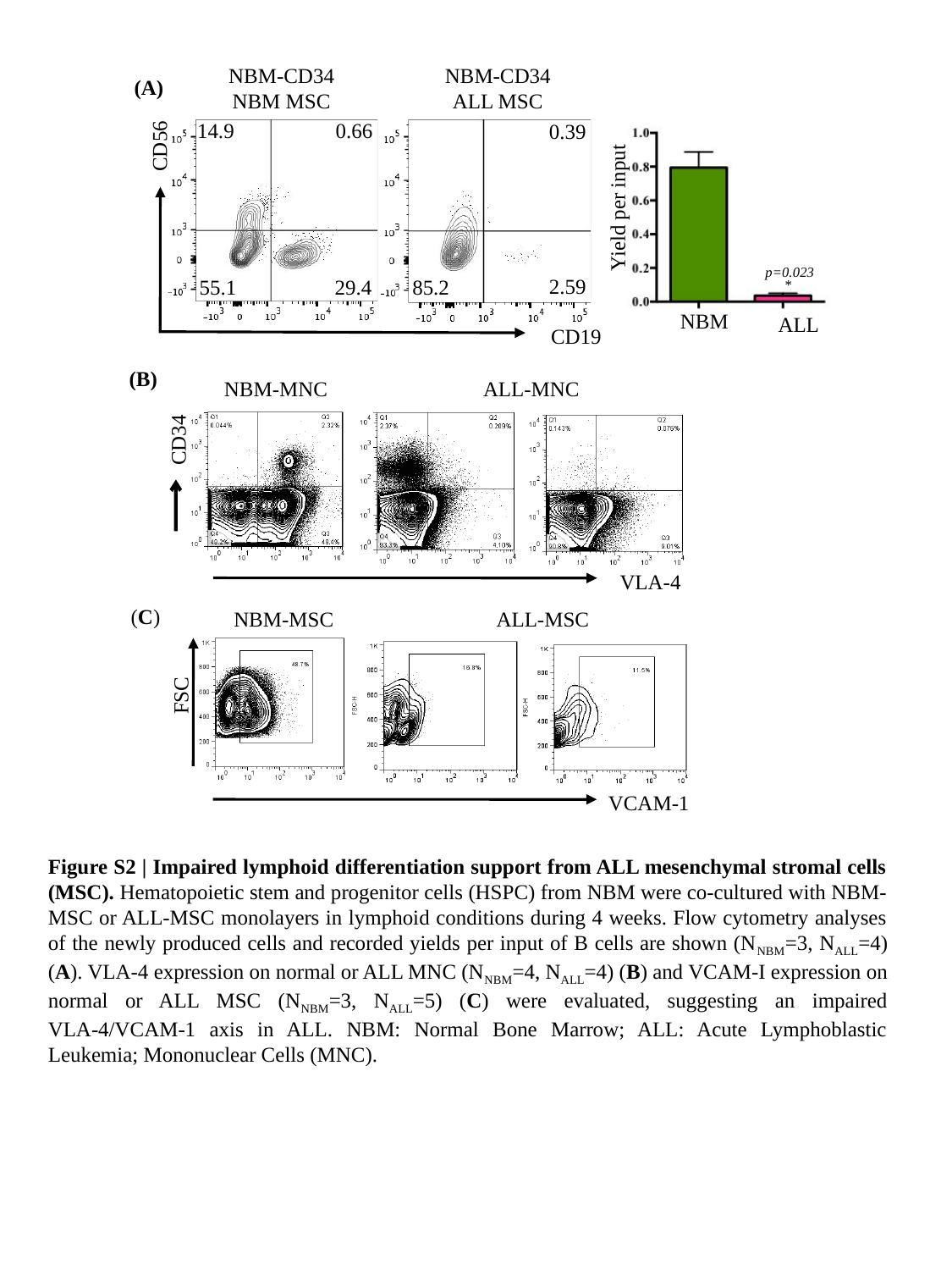

NBM-CD34
ALL MSC
NBM-CD34
NBM MSC
14.9
0.66
0.39
2.59
29.4
85.2
55.1
NBM
CD56
Yield per input
p=0.023
*
ALL
CD19
(A)
(B)
NBM-MNC
ALL-MNC
CD34
VLA-4
(C)
NBM-MSC
ALL-MSC
FSC
VCAM-1
Figure S2 | Impaired lymphoid differentiation support from ALL mesenchymal stromal cells (MSC). Hematopoietic stem and progenitor cells (HSPC) from NBM were co-cultured with NBM-MSC or ALL-MSC monolayers in lymphoid conditions during 4 weeks. Flow cytometry analyses of the newly produced cells and recorded yields per input of B cells are shown (NNBM=3, NALL=4) (A). VLA-4 expression on normal or ALL MNC (NNBM=4, NALL=4) (B) and VCAM-I expression on normal or ALL MSC (NNBM=3, NALL=5) (C) were evaluated, suggesting an impaired VLA-4/VCAM-1 axis in ALL. NBM: Normal Bone Marrow; ALL: Acute Lymphoblastic Leukemia; Mononuclear Cells (MNC).
