# Supplementary material for: Pro-inflammatory-Related Loss of CXCL12 Niche Promotes Acute Lymphoblastic Leukemic Progression at the Expense of Normal Lymphopoiesis
Source: Front Immunol. 2017 Jan 5;7:666. doi: 10.3389/fimmu.2016.00666 (PMC5216624; doi:10.3389/fimmu.2016.00666)
Supplement: Supplementary file 4 [file Presentation_3.ppt]

## Slide 1
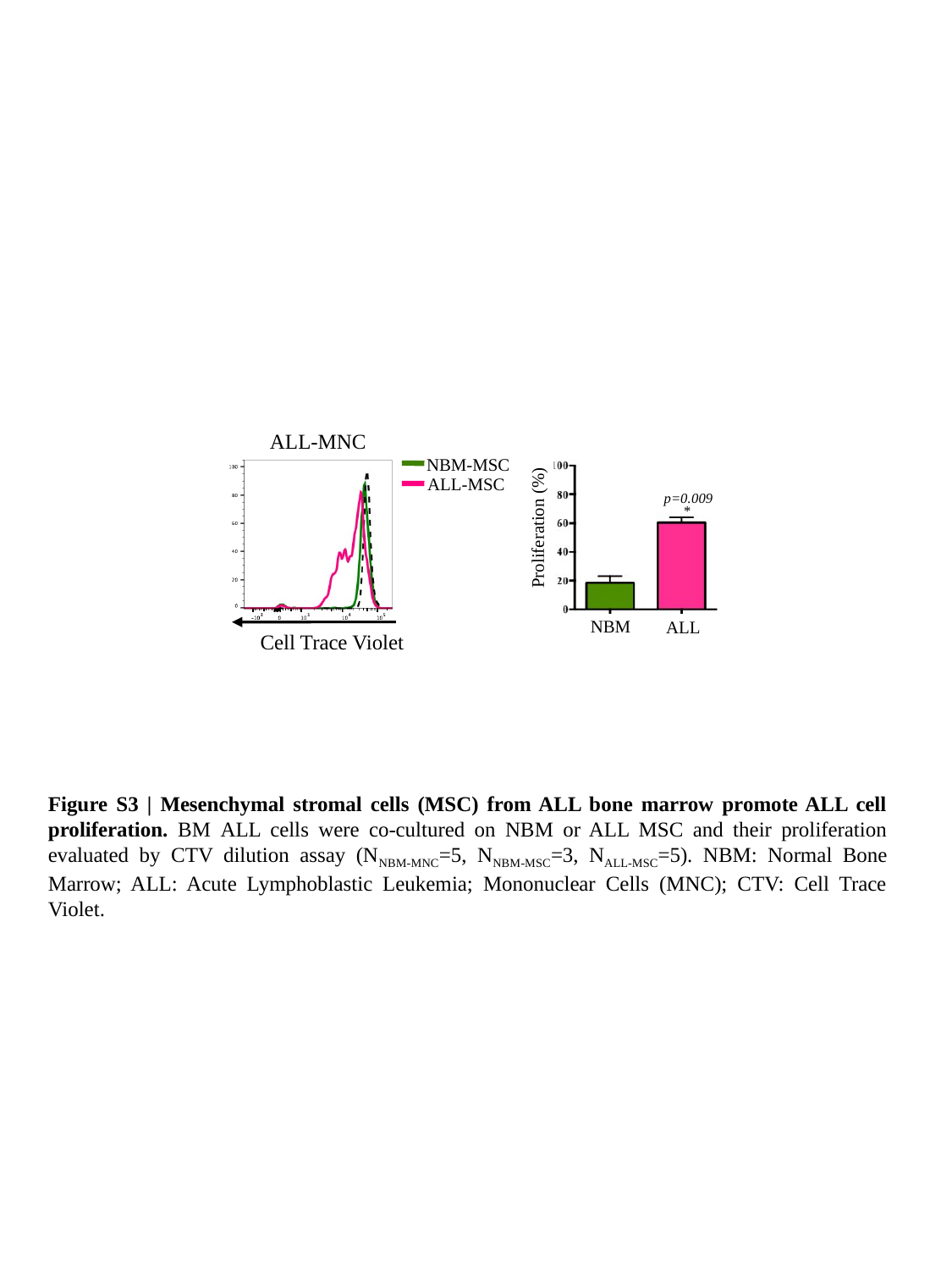

ALL-MNC
Cell Trace Violet
NBM-MSC
ALL-MSC
p=0.009
*
Proliferation (%)
NBM
ALL
Figure S3 | Mesenchymal stromal cells (MSC) from ALL bone marrow promote ALL cell proliferation. BM ALL cells were co-cultured on NBM or ALL MSC and their proliferation evaluated by CTV dilution assay (NNBM-MNC=5, NNBM-MSC=3, NALL-MSC=5). NBM: Normal Bone Marrow; ALL: Acute Lymphoblastic Leukemia; Mononuclear Cells (MNC); CTV: Cell Trace Violet.
