# Supplementary material for: Pro-inflammatory-Related Loss of CXCL12 Niche Promotes Acute Lymphoblastic Leukemic Progression at the Expense of Normal Lymphopoiesis
Source: Front Immunol. 2017 Jan 5;7:666. doi: 10.3389/fimmu.2016.00666 (PMC5216624; doi:10.3389/fimmu.2016.00666)
Supplement: Supplementary file 5 [file Presentation_4.ppt]

## Slide 1
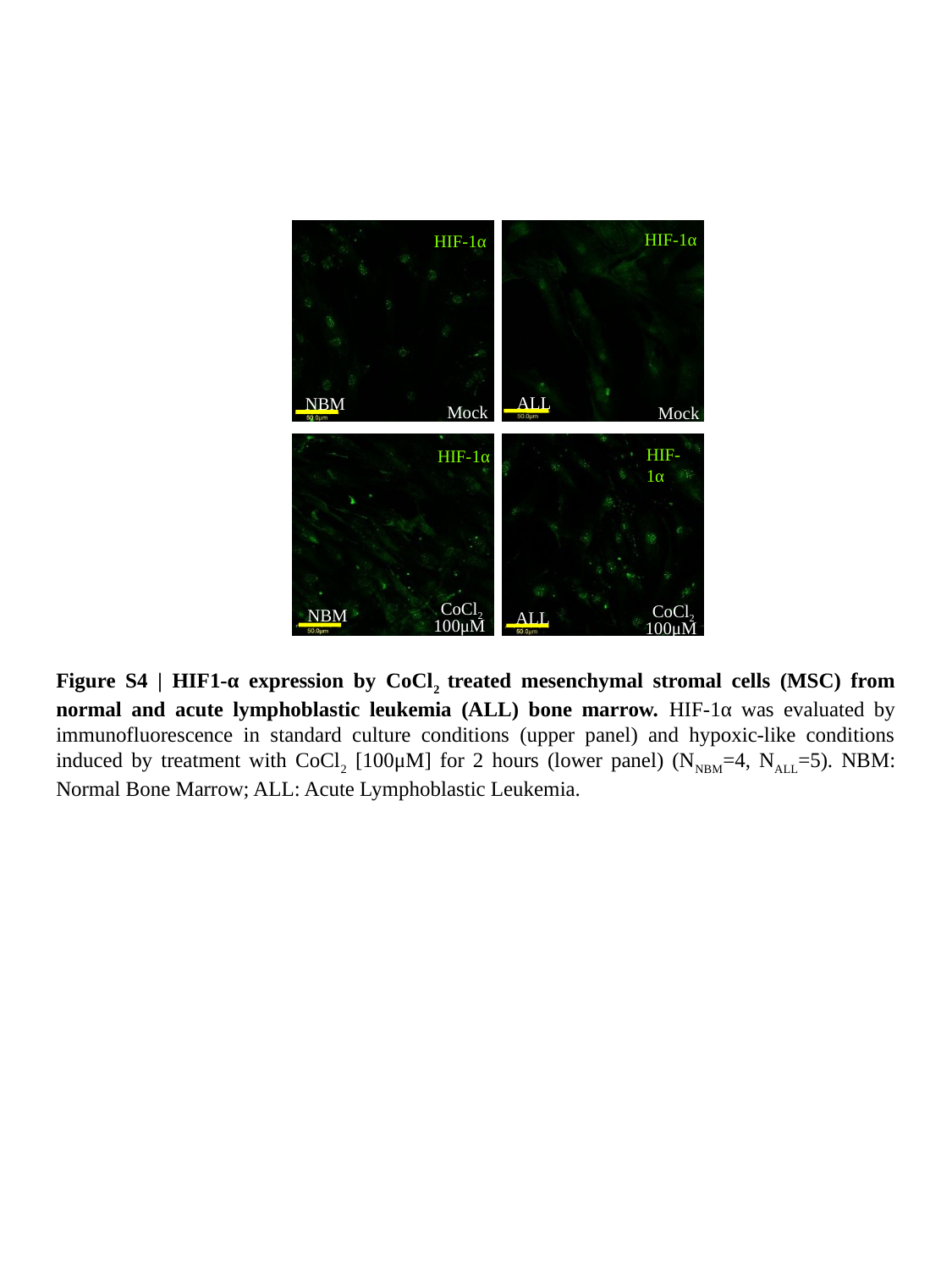

HIF-1α
HIF-1α
ALL
NBM
Mock
Mock
HIF-1α
HIF-1α
CoCl2
100μM
CoCl2
100μM
NBM
ALL
Figure S4 | HIF1-α expression by CoCl2 treated mesenchymal stromal cells (MSC) from normal and acute lymphoblastic leukemia (ALL) bone marrow. HIF-1α was evaluated by immunofluorescence in standard culture conditions (upper panel) and hypoxic-like conditions induced by treatment with CoCl2 [100μM] for 2 hours (lower panel) (NNBM=4, NALL=5). NBM: Normal Bone Marrow; ALL: Acute Lymphoblastic Leukemia.
