# Supplementary material for: Pro-inflammatory-Related Loss of CXCL12 Niche Promotes Acute Lymphoblastic Leukemic Progression at the Expense of Normal Lymphopoiesis
Source: Front Immunol. 2017 Jan 5;7:666. doi: 10.3389/fimmu.2016.00666 (PMC5216624; doi:10.3389/fimmu.2016.00666)
Supplement: Supplementary file 6 [file Presentation_5.ppt]

## Slide 1
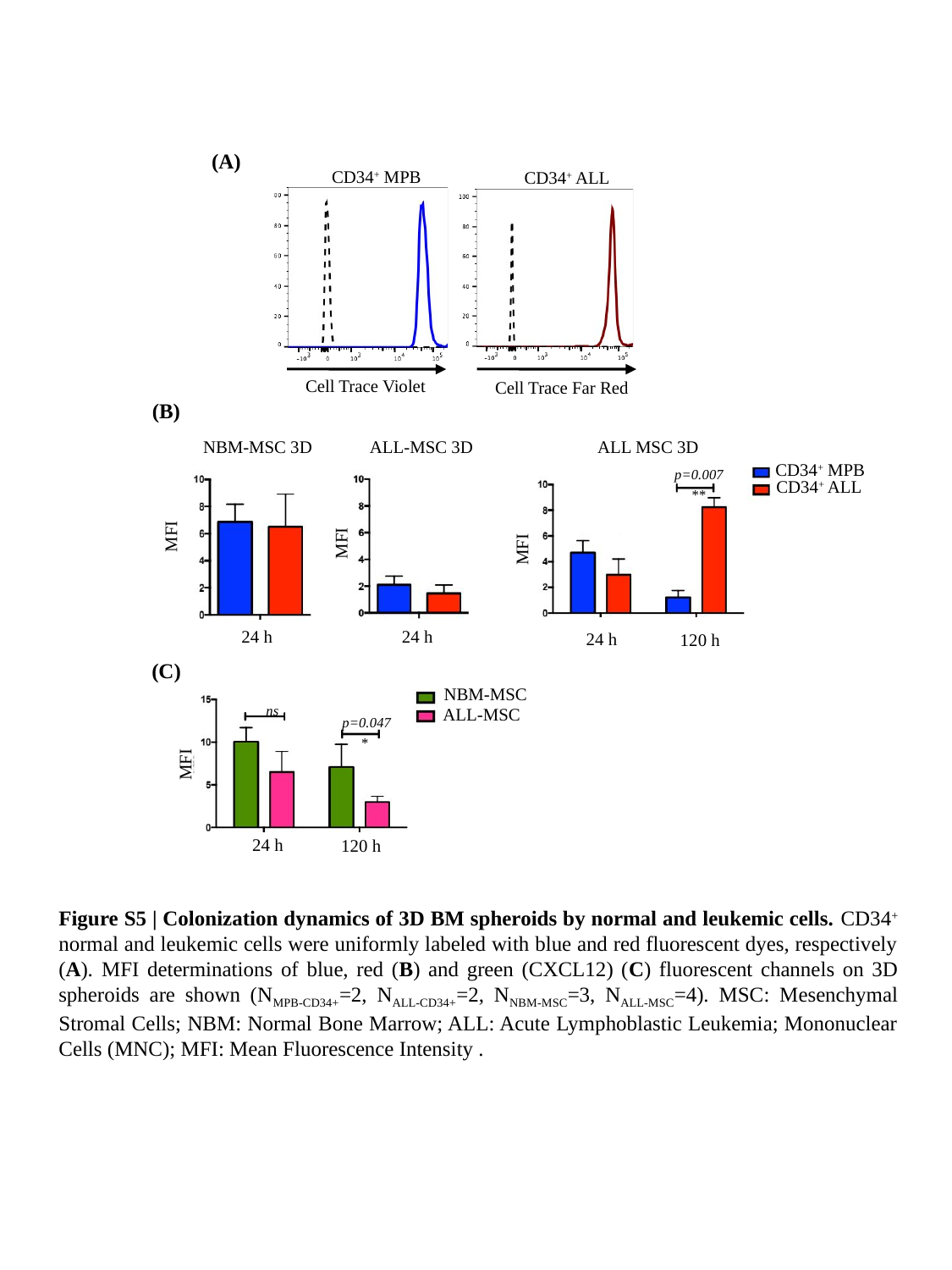

(A)
CD34+ MPB
CD34+ ALL
Cell Trace Violet
Cell Trace Far Red
(B)
ALL MSC 3D
NBM-MSC 3D
ALL-MSC 3D
p=0.007
CD34+ MPB
**
CD34+ ALL
MFI
MFI
MFI
24 h
24 h
24 h
120 h
(C)
NBM-MSC
ALL-MSC
ns
MFI
24 h
120 h
p=0.047
*
Figure S5 | Colonization dynamics of 3D BM spheroids by normal and leukemic cells. CD34+ normal and leukemic cells were uniformly labeled with blue and red fluorescent dyes, respectively (A). MFI determinations of blue, red (B) and green (CXCL12) (C) fluorescent channels on 3D spheroids are shown (NMPB-CD34+=2, NALL-CD34+=2, NNBM-MSC=3, NALL-MSC=4). MSC: Mesenchymal Stromal Cells; NBM: Normal Bone Marrow; ALL: Acute Lymphoblastic Leukemia; Mononuclear Cells (MNC); MFI: Mean Fluorescence Intensity .
